# Harnessing mechanical cues in the cellular microenvironment for bone regeneration

**DOI:** 10.3389/fphys.2023.1232698

**Published:** 2023-10-09

**Authors:** Timothy O. Josephson, Elise F. Morgan

**Affiliations:** ^1^ Biomedical Engineering, Boston University, Boston, MA, United States; ^2^ Center for Multiscale and Translational Mechanobiology, Boston University, Boston, MA, United States; ^3^ Mechanical Engineering, Boston University, Boston, MA, United States

**Keywords:** bone, mechanobiology, marrow stromal cells (MSCs), mechanical microenvironment, osteogenesis

## Abstract

At the macroscale, bones experience a variety of compressive and tensile loads, and these loads cause deformations of the cortical and trabecular microstructure. These deformations produce a variety of stimuli in the cellular microenvironment that can influence the differentiation of marrow stromal cells (MSCs) and the activity of cells of the MSC lineage, including osteoblasts, osteocytes, and chondrocytes. Mechanotransduction, or conversion of mechanical stimuli to biochemical and biological signals, is thus part of a multiscale mechanobiological process that drives bone modeling, remodeling, fracture healing, and implant osseointegration. Despite strong evidence of the influence of a variety of mechanical cues, and multiple paradigms proposed to explain the influence of these cues on tissue growth and differentiation, even a working understanding of how skeletal cells respond to the complex combinations of stimuli in their microenvironments remains elusive. This review covers the current understanding of what types of microenvironmental mechanical cues MSCs respond to and what is known about how they respond in the presence of multiple such cues. We argue that in order to realize the vast potential for harnessing the cellular microenvironment for the enhancement of bone regeneration, additional investigations of how combinations of mechanical cues influence bone regeneration are needed.

## 1 Introduction

The response of skeletal cells to mechanical stimuli is fundamental to understanding, treating, and preventing orthopaedic injuries and diseases. The fact that bone is responsive to mechanical stimulation is well documented: athletes whose bodies experience more intense loading have increased bone mass (Bennell et al., 1997), while astronauts lose bone mass after spending time in low-gravity environments (Orwoll et al., 2013). Distraction osteogenesis, a surgical process of lengthening and reshaping a bone, improves healing outcomes in treatment of non-union by providing controlled levels of mechanical stimulation (Kanellopoulos and Soucacos, 2006; Fu et al., 2021), while metal implants can locally weaken bone due to stress shielding (Sumner, 2015; Augat and von Rüden, 2018). Mechanical cues also have a strong influence on the outcomes of fracture healing (Augat et al., 2021) and implant osseointegration (Mavrogenis et al., 2009). Bone responds to mechanical cues through multiple mechanisms, including osteocyte signaling, which plays an importantrole in bone remodeling, and the differentiation of MSCs. In the context of bone regeneration, mechanobiologically driven differentiation of MSCs is particularly important, as it determines the cell and tissue types that will form as a fracture heals. There is a long standing belief that the mechanoresponsiveness of bone, if well understood, could be routinely harnessed for therapeutic benefit in many clinical contexts. Indeed, in the words of Julius Wolff more than a century ago, “the remodelling force is a therapeutic force of immeasurable magnitude” (Wolff, 1986).

**FIGURE 1:**
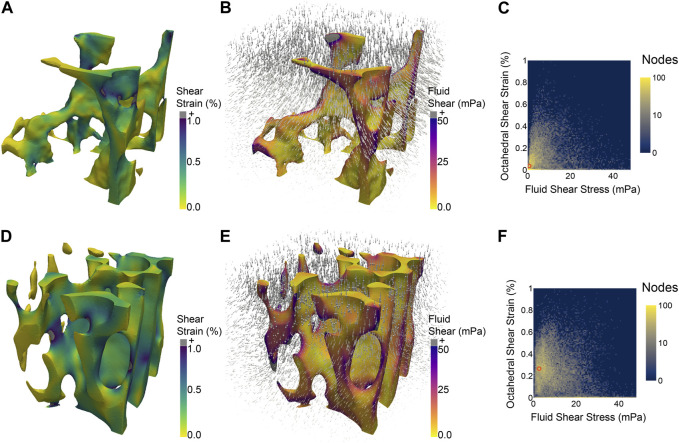
Two regions of trabecular bone from the same mouse vertebra, each subjected to the same simulated macroscale stimulus (uniaxial compression of 2,500 *μϵ*) experience different distributions of microscale stimuli, such as octahedral shear strain **(A,D)** induced by the applied compression and fluid shear stress **(B,E)** due to the flow of marrow induced by the compression. 2D histograms **(C,F)** illustrate the difference in distributions of shear strain and fluid shear stress between the two regions of bone. The most prevalent combination of the two micro-stimuli in each region is denoted by an orange circle at (0.75 mPa, 0.035%) **(C)** and (2.75 mPa, 0.265%) **(F)**.

The field of orthopaedics has sought to translate this therapeutic force in a variety of ways to enhance bone regeneration. As reviewed by Mavčič and Antolič (2012), Augat et al. (2021), and Huang et al. (2013), numerous studies have attempted to enhance healing by regulating the magnitude and frequency of loading at different stages of the healing process. While some studies have achieved promising results, these results have yet to be generalized to actionable guidelines for other scenarios, or even other patients, due to the complex dependence of the mechanical stimuli on parameters such as fracture geometry and location, as well as to other factors such as patient age and co-morbidities. The first of these causes—the complexity of the relevant mechanics—arises from the fact that similar loading of bones at the macroscale may result in distinctly different microenvironmental stimuli in different patients, in different regions of bone (Figure 1) and over time as the tissue microstructure changes with adaptation. When bones experience forces, whether through load bearing or muscle contraction, the cortical and trabecular microstructures deform. The same is true for the soft tissues and woven bone that form in the initial and intermediate stages of fracture healing, and in the periosteum (McBride et al., 2011). These deformations push and pull on the cells residing within the complex geometries of bone tissue (Vaughan et al., 2015) and drive the flow of marrow and extracellular fluid around cells (Metzger et al., 2015). These local stimuli—tissue strains, fluid-based stresses, and geometric cues—constitute the cellular mechanical microenvironment.

Hence, in order to understand how and why bones adapt and heal in the ways that they do, focus has shifted from the macroscale stimuli that whole bones receive to the microscale stimuli that skeletal cells experience. However, the relative influence and the optimal levels of the various microenvironmental stimuli are not well known, particularly in complex microenvironments with a variety of different stimuli. By reviewing the evidence for the influence of these specific stimuli, individually and in combination, on MSC differentiation and the prevailing theories of how combinations of them act to regulate bone regeneration, we aim to demonstrate the potential for further harnessing the mechanical microenvironment and to identify the critical questions that must be answered in order to do so. While this review will primarily focus on the microenvironmental stimuli that influence osteogenic differentiation of MSCs in the context of bone regeneration following trauma, chondrogenic differentiation of MSCs and chondrocyte-to-osteoblast transdifferentiation also play important roles in endochondral ossification and can both be regulated by many of the same types of microenvironmental stimuli as are discussed for osteogenic differentiation of MSCs (Wong et al., 2018; McDermott et al., 2019).

## 2 Individual microenvironmental stimuli

### 2.1 Exogenous mechanical stimulation

Exogenous stimuli are those induced by applied mechanical loads. These stimuli arise from both the solid and fluid compartments of bone, and include solid strain, fluid shear stress, and hydrostatic pressure. Applied loads are often transient in nature, so the microenvironment is characterized also by the frequency and loading history of these exogenous stimuli, not just the instantaneous magnitudes.

A large body of work provides evidence [as reviewed by Scott et al. (2008) and Steward and Kelly (2015)] that cyclic strains can influence MSC differentiation and that the magnitude and frequency of loading are relevant factors. MSCs are capable of sensing both tensile and compressive strains, and they respond to the two in distinct ways. Cyclic tensile strains have been frequently associated with osteogenic differentiation of MSCs on both 2D substrates (Qi et al., 2008; Kearney et al., 2010) and 3D soft scaffolds (Haudenschild et al., 2009; Yu et al., 2021) while cyclic compression of MSCs in 3D constructs has been shown to promote both osteogenesis (Schreivogel et al., 2019) and chondrogenesis (Pelaez et al., 2009). Additionally, stretch-activated cation channels (SACC) have been implicated as an important component of the response to tissue strain-based deformations of cells and have been associated with synthesis of both glycosaminoglycans [GAGs, McMahon et al. (2008)] and collagen I (Kearney et al., 2010). The synthesis of matrix components as downstream effect of mechanical stimulation is a main mechanism by which the mechano-responsiveness of MSCs and osteoblasts are regulated, as changes in the matrix will likely modulate the cellular microenvironment. This general type of regulatory loop is referred to as mechanomics (Knothe Tate et al., 2016). Haudenschild et al. (2009) identified that *α*- and *β*-catenin, which are relevant in cytoskeletal mechanics and the osteogenically important Wnt signaling pathway, are regulated differently by the type of strain, with *α*-catenin upregulated by compressive strains and *β*-catenin upregulated by tensile/distortional stretch.

Despite these findings, the optimal strain magnitudes and frequencies for promoting osteogenesis are difficult to ascertain in a broadly applicable manner. This can be attributed to two main causes: differences in experimental setups and measured outputs, and the presence of other stimuli that are not always accounted for. Studies often use different loading conditions (e.g., uniaxial, biaxial, bending-based stretch), different culture conditions (e.g., serum vs. serum-free media), study different cell types (e.g., MSCs, osteoblasts, osteoblast-like cells), and measure the expression of different osteogenic markers (e.g., RUNX2, osteopontin, osteocalcin). These differences make quantitative agreement and reproducibility across studies difficult to assess. The presence of confounding factors amplifies this challenge. Differences among substrates in regards to other cues that cells experience, such as stiffness and/or curvature present another barrier to comparing results across studies. Additionally, macroscale tensile or compressive loading of 3D structures like bone can easily result in cells experiencing combinations of both tension and compression at the microscale (Niebur et al., 2000; Fields et al., 2010). Further, the fact that cells exist within aqueous environments means that strain-based stimulation doesn’t occur independently of fluid-based stimuli; however, the latter are typically not accounted for when examining the influence of strain.

A variety of studies have emphasized the importance of fluid shear stress, due to both oscillatory flow and continuous unidirectional flow, in skeletal cell mechanobiology. Arnsdorf et al. (2009) demonstrated that oscillatory fluid flow promotes osteogenic differentiation of MSCs by activating RhoA, a regulator of ROCKII and subsequently cytoskeletal tension and organization. Corrigan et al. (2018) found that calcium channel transient receptor potential subfamily V member 4 (TRPV4) is critical for flow-based mechanotransduction in MSCs and is strongly associated with mechanosensitivity of the primary cilium. A variety of studies have demonstrated that parallel flow over a flat monolayer of MSCs can induce osteogenic behavior when the shear stress is on the order of 1 Pa in constant (Reich and Frangos, 1991; Yourek et al., 2010) and oscillatory (Li et al., 2004; Stavenschi et al., 2017) flow conditions, with both the magnitude and frequency of flow being influential factors (Stavenschi et al., 2017). Interestingly, studies that examine flow through 3D scaffolds report that much lower shear stresses, on the order of 1 mPa, are associated with increased osteogenesis while stresses above approximately 10 mPa are detrimental to cell viability (Porter et al., 2005; Melke et al., 2018). This discrepancy may be indicative of a broader difference between cell-microenvironment interactions in 2D vs. 3D contexts (Baker and Chen, 2012). In addition to regulation of MSC differentiation, fluid shear stress has been shown to induce immunomodulatory behavior in MSCs (Skibber et al., 2022). Along with changes in fluid shear stress, fluid flow is also associated with changes in hydrostatic pressure. High cyclically applied hydrostatic pressure (∼100–1,000 kPa) has been associated with chondrogenic differentiation of MSCs (Wagner et al., 2008; Stavenschi and Hoey, 2019) while lower pressures (∼10–300 kPa) have been associated with osteogenesis (Burger et al., 1992; Tang et al., 2017; Reinwald and El Haj, 2018).

Not only does fluid flow apply forces to cells, it also distributes nutrients. The flow of nutrient- and oxygen-carrying fluid helps to ensure the distribution of nutrients to cells throughout a 3D environment to maintain cell viability and enable proliferation and differentiation (Karande et al., 2004; Amini and Nukavarapu, 2014). Donahue et al. (2003) found that cells were significantly less responsive to shear stress in nutrient-free media, further demonstrating the difficulty of separating the effects of chemotransport and fluid flow.

### 2.2 Endogenous cues in the mechanical microenvironment

In the absence of externally applied loads, there are still physical cues endogenous to the microenvironment. These factors, which include curvature as well as matrix/substrate stiffness, influence cell adhesion and cytoskeletal tension and are capable of driving MSC differentiation.

As reviewed by Werner et al. (2020), cells are capable of sensing curvatures at both a subcellular length scale (primarily through focal adhesion placement and growth) and length scales greater than or equal to the size of the cell (due to interactions between stress fibers and the nucleus). For MSCs cultured on hemispherical concave and convex surfaces, Werner et al. (2017) found that convex curvatures increased osteogenic gene expression while concave curvatures increased cell migration speeds. Werner et al. (2019) additionally introduced the notion of direction-dependent “perceived curvature” on non-spherical anisotropic curvatures (such as a cylindrical curvature). The perceived curvature acknowledges that cells oriented along the long axis of a cylinder experience a different amount of curvature and therefore undergo less bending than cells oriented perpendicular to the long axis; MSCs were observed to alter their migration behavior, ostensibly to avoid this bending. Callens et al. (2023) studied pre-osteoblasts on patterned substrates with a broader range of curvatures and found that groups of cells preferentially pattern surfaces with at least one negative principal curvature (i.e., concave-saddle), though over time groups of cells are able overcome convexities through cell-cell interactions that result in the formation of cell sheets that bridge unfavorable curvatures. Yang et al. (2022) found that saddle-like surfaces (triply periodic minimal surface-based scaffolds) promoted both osteogenesis and angiogenesis *in vivo*. The magnitude of curvature, in addition to the shape of curvature, is relevant to MSCs, with Swanson et al. (2022) finding that small spherical pores with curvatures in the range (16.0, 33.3) mm^−1^ maintained the stemness of MSCs while larger pores with curvatures in the range (4.7, 8.0) mm^−1^ promoted osteogenic differentiation, however there is a lack of thorough examination of how variations in curvature magnitude influence osteogenic behavior of MSCs, particularly when coupled with variations in curvature shape. The lack of consensus as to what constitutes an “optimal” curvature for osteogenesis may be attributable to the possibility that different processes are stimulated by different curvatures, for example, a curvature that promotes osteogenic differentiation may differ from a curvature that promotes tissue formation.

The curvature of a surface influences how cells attach to it, making surface curvature an important determinant of cell shape (Werner et al., 2017). McBeath et al. (2004) found that cell shape regulates RhoA, which in turn regulates the switch between adipogenic and osteogenic differentiation, with cells becoming osteoblasts when they were allowed to spread, and adipocytes when they were maintained as round. Similarly, Kilian et al. (2010) found that seeding MSCs on 2D islands of different shapes led to different lineage commitments, with shapes that had higher aspect ratios or concave subcellular curvatures generally found to increase cytoskeletal contractility and osteogenic differentiation. This association among curvature, cell shape, and the cytoskeleton emphasizes the mechanical nature of sensing of microenvironmental curvature. Through focal adhesions and cytoskeletal mechanics, MSCs are also able to sense the local stiffness of their microenvironment. When seeded on substrates of varying stiffnesses, MSCs were found to undergo morphological changes and exhibited neurogenic, myogenic, or osteogenic differentiation depending on substrate stiffness, further implicating cytoskeletal contractility as a key sensory mechanism of the physical microenvironment (Engler et al., 2006). There has been extensive study of the role of matrix/substrate stiffness and the interactions between MSCs and the extracellular matrix more generally, which have been reviewed by Assis-Ribas et al. (2018) and Lv et al. (2015).

### 2.3 Other factors

There are, of course, many non-mechanical factors that influence osteogenesis and bone regeneration. As reviewed by Hayrapetyan et al. (2015), there are various hormones, cytokines, and signaling pathways that are critical to osteogenic differentiation. Bone regeneration is also strongly coupled to other physiological processes, including angiogenesis which delivers oxygen and essential nutrients [as reviewed by Kanczler and Oreffo (2008)], the immune/inflammatory response which plays an essential role in initiating the repair process [as reviewed by Claes et al. (2012)], and the presence of extracellular matrix proteins which have also been shown to play a significant role in mediating the behavior of MSCs (Datta et al., 2006). The influence of nutrients can be particularly relevant *in vitro*, as the choice of media can strongly influence the differentiation of MSCs (Ho et al., 2011; Kyllönen et al., 2013). While these chemical and biological factors are indeed essential to the bone regeneration process, they too occur within the context of a mechanical environment and are coupled with the mechanobiological response of skeletal cells.

## 3 Combinations of stimuli in the mechanical microenvironment

The simultaneous presence of multiple stimuli in the microenvironments of skeletal cells *in vivo* (Moraes et al., 2011) makes it difficult to draw general conclusions about skeletal mechanobiology from examining only individual stimuli. Relatively few studies have attempted to directly quantify the effects of multiple mechanical cues acting concurrently, leaving open questions about how cells respond to combinations of cues.

Jiao et al. (2022) examined the synergistic effects of adhesion morphology and fluid shear stress by applying different levels of flow to cells seeded on differently shaped micro patterned substrates. Their results demonstrated that fluid shear stress and adhesion morphology could work cooperatively or antagonistically to regulate osteogenesis, with osteogenically favorable adhesion morphologies enhancing the osteogenic response induced by fluid flow and unfavorable morphologies blunting its influence. Further, they found that fluid shear stress had no effect on cell shape or spreading, indicating that the two cues regulate osteogenesis through different mechanisms.

Additional studies on the influence of multiple concurrent mechanical cues are based on observation of tissue differentiation *in vivo*, often in the context of bone regeneration following fracture. These studies have formulated hypotheses of how mechanical stimuli lead to local tissue differentiation into bone, cartilage, or fibrous tissue. Different models have considered different pairings of tensile, compressive, and shear stresses and strains as well as fluid flow velocities and hydrostatic pressures. Prendergast et al. (1997), Carter et al. (1998), and Claes and Heigele (1999) each proposed paradigms to predict tissue differentiation within a healing fracture callus. A comparative analysis by Isaksson et al. (2006a) between the mechanoregulatory models of Carter et al. (1998), Claes and Heigele (1999), Lacroix and Prendergast (2002), and a model based on deviatoric strain by Isaksson et al. (2006b) and found that the model by Lacroix and Prendergast (2002), which is an extension of the model proposed by Prendergast et al. (1997) and postulates that tissue differentiation depends on combinations of shear strain and fluid flow velocity, was the most consistent with experimental data, matching those data in most, but not all, cases that were examined. Song et al. (2012) examined similar relationships between MSC lineage commitment and stress and strain by measuring local cellular deformations and the expression of lineage-associated genes. Despite efforts towards a working mechanobiological theory of bone regeneration, more work is needed to fully unify the influence of the various endogenous, exogenous, and non-mechanical factors into a robust and clinically translatable predictive model.

All of the aforementioned models consider how combinations of stimuli impact cell and tissue differentiation in regions such as a fracture callus where there is preliminary granulation tissue present. A different class of mechanobiological models considers how various cues promote the growth of new tissue into pore space, which is relevant in both bone remodeling and the osseointegration of bone tissue engineering scaffolds and other implants. A series of models by Geris and colleagues (Guyot et al., 2014; Guyot et al., 2016; Mehrian et al., 2018) predicts neotissue growth due to curvature, fluid shear stress, and metabolic factors (oxygen, glucose, pH) for scaffolds in perfusion bioreactors. Another growth model considers the growth and remodeling of trabecular bone (Aland et al., 2020) using both strain energy density and volumetric compression as possible strain-based remodeling stimuli. Both types of models describe important parts of the mechanobiological response of bone, but neither captures its full scope.

## 4 Discussion

The cellular microenvironment contains a variety of mechanical stimuli that both individually and collectively appear to regulate cellular activity and mediate osteogenesis during bone repair and regeneration. While the fact that these stimuli, including curvature, stiffness, strain, fluid shear stress, and hydrostatic pressure, are influential has been convincingly demonstrated, a thorough quantitative understanding of their influence at various magnitudes, frequencies, and durations is still lacking, particularly when multiple stimuli act concurrently. Such an understanding could enable new therapies for enhanced bone regeneration, patient-specific treatment plans, and improved design of orthopaedic implants. In the absence of that understanding, progress on these fronts is likely to be slow.

Mechanobiological considerations are increasingly being made in the treatment of bone fractures, as well as injuries in other tissues, as described for example, by the revised definition of the term “mechanotherapy” (Huang et al., 2013; Thompson et al., 2016). Several methods have been proposed to provide controlled levels of mechanical stimulation to skeletal cells, including low intensity vibration (LIV) and low intensity pulsed ultrasound (LIPUS), which have in at least some studies shown a potential for improving bone regeneration (Thompson et al., 2016); however, recent studies have questioned the efficacy of these treatments and called for additional investigation into the situations when such treatments may provide benefit, further demonstrating the need for a thorough understanding of how MSCs respond to microenvironmental stimuli (Lou et al., 2017; Searle et al., 2023). Using the mechanobiological model of Prendergast et al. (1997) and Miramini et al. (2015) demonstrated that different locking compression plate configurations can yield different mechanical microenvironments and tissue differentiation patterns, highlighting the potential of using enhanced understanding of mechanical microenvironments to impact specific approaches to fracture fixation.

Patient-specific treatments are particularly relevant in the context of aging-related changes to bone. The mechanoresponsiveness of bone has been shown to be altered by aging in both animals (Turner et al., 1995) and humans (Kohrt, 2001), though it remains unclear whether this alteration is due to diminished mechanosensitivity of cells or to microstructural changes that alter the microenvironmental stimuli that they receive. Furthering the understanding of both the microstructural changes associated with aging and the effects of aging on cell mechanoresponsiveness could support the development of treatments and activity guidelines for both improved fracture healing and maintenance of bone mass that are specific to an individual’s age and health.

In surgical situations that call for the use of orthopaedic implants, the design of those implants offers an opportunity to apply the understanding of skeletal mechanobiology to custom-designed microenvironments. Among the most direct possible applications is in the design and development of bone tissue engineering scaffolds. Microenvironmentally-informed scaffold architectures could be used to regulate the stimuli that cells seeded on their surfaces perceive in order to enhance tissue growth. This could lead to the development of artificial bone grafts that are both safer and more effective than auto- or allografts. Other relevant applications include improving the osseointegration of joint replacement implants and developing fracture fixation implants that regulate the allowable motion of a fracture site to improve healing outcomes.

In order to fully realize the potential of harnessing the mechanical microenvironment, further work is needed. Future studies that assess the influence of individual or combinations of stimuli should quantify both the applied macroscale stimuli as well as the local microscale stimuli. This broader accounting of stimuli would enhance the applicability of findings and make them relevant beyond the scope of particular experimental setups. Additionally, efforts should be made to account for stimuli beyond those being directly investigated, for example, compression-induced fluid flow. To this end, computational simulation offers a powerful tool for analysis of microenvironments (Figure 1) that can be paired with experimental results (Schulte et al., 2013). Another step to improving the robustness and generalizability of studies examining the effect of microenvironmental stimuli on osteogenesis is to examine multiple markers of osteogenic differentiation, with an eye towards developing a minimum standard set of readouts. Osteocalcin, osteopontin, and RUNX2 are all commonly used markers of osteogenic differentiation; however many studies examine only one, making it difficult to compare results between studies.

Overall, the importance of mechanical cues to bone regeneration highlights the importance of elucidating the mechanoresponsiveness of skeletal cells to combinations of microenvironmental stimuli. Doing so has the potential to address a variety of key clinical needs and answer major questions about the nature of skeletal mechanobiology.
